# Distinct Profiles of Patient-Reported Outcomes Across Allergen Signatures in Chronic Rhinosinusitis

**DOI:** 10.3390/life15121835

**Published:** 2025-11-28

**Authors:** Dachan Kim, Chan Min Jung, Hyung-Ju Cho, Chang-Hoon Kim, Min-Seok Rha

**Affiliations:** 1 Department of Otorhinolaryngology, Yonsei University College of Medicine, 50-1 Yonsei-ro, Seodaemun-gu, Seoul 03722, Republic of Korea; 2 Department of Otorhinolaryngology, Gangnam Severance Hostpital, Yonsei University College of Medicine, Seoul 06273, Republic of Korea; 3 The Airway Mucus Institute, Yonsei University College of Medicine, Severance Hospital, Seoul 03722, Republic of Korea

**Keywords:** chronic rhinosinusitis, allergen signatures, nonnegative matrix factorization, patient-reported outcome measures

## Abstract

Background: Chronic rhinosinusitis (CRS) exhibits marked symptom heterogeneity that is not fully explained by anatomy or endotypes. Although allergen types shape symptom patterns in allergic rhinitis, largescale systematic analyses linking allergen sensitization profiles to patient-reported outcome measures in patients with CRS are limited. Methods: We conducted a multicenter, retrospective surgical cohort study (*n* = 1880) including patients with CRS who underwent preoperative specific IgE testing for 35 inhalant allergens and completed the 22-item Sino-Nasal Outcome Test (SNOT-22) questionnaire within 1 year. Using a previously validated nonnegative matrix factorization model, we deconvolved each patient’s IgE profile into four allergen signatures (Mite, Grass/Weed, Pet, and Tree) and defined a dominant group. Associations between signature contributions and SNOT-22 items, domain subscores, and total score were estimated by ordinary least squares, adjusting for age, sex, nasal polyps, and asthma, with coefficients scaled per 10-percentage-point increase. Item-level multiplicity was controlled for using the false discovery rate. Seasonality was assessed using monthly means and the coefficient of variation of the dominant group. Results: Dominant groups were nonallergic (50%), mite (26%), grass/weed (9%), pet (9%), and tree (5%). Symptoms varied by age and sex, characterized by notably low nasal scores with aging and a high female burden for several items, motivating covariate adjustment. Signature–symptom associations were domain-specific: the pet signature showed the strongest and most consistent associations with nasal domain (such as rhinorrhea and nasal obstruction) and emotion domain (feelings of embarrassment); mite and grass/weed signatures were linked to the function domain (daytime fatigue/productivity); whereas the tree signature showed no significant associations. Seasonal patterns aligned with exposure ecology: grass/weed and tree groups had the largest relative variation (high coefficient of variations), the pet group showed the highest absolute burden year-round, and the mite group varied modestly with winter–spring predominance. Conclusions: Allergen signatures distilled from routine IgE panels explained meaningful variations in CRS patient-reported outcome measures, mapping to distinct symptom domains and seasonal profiles. Incorporating signature information into clinical assessments may support personalized counseling, anticipatory management around exposure windows, and targeted evaluation of environmental or immunologic interventions.

## 1. Introduction

Chronic rhinosinusitis (CRS) is a prevalent, complex inflammatory disorder of the nasal cavity and paranasal sinuses that imposes a substantial health care burden [1]. CRS also impairs patients’ quality of life through persistent nasal congestion, rhinorrhea, facial pain, and smell loss, with downstream effects on sleep, productivity, and psychological wellbeing [2,3]. Patient-reported outcome measures (PROMs) are indispensable for capturing disease impact and monitoring treatment response in both clinical care and research [4]. The 22-item Sino-Nasal Outcome Test (SNOT-22) is the gold-standard, disease-specific PROM for CRS, covering five validated domains—Nasal, Ear/Facial, Sleep, Function, and Emotion—and demonstrating excellent psychometric performance and routine use in CRS cohorts [5,6]. However, patients with CRS exhibit marked symptom heterogeneity, even with similar endoscopic or radiographic findings, reflecting underlying immunologic diversity [7].

Allergen sensitization patterns shape symptom expression in allergic rhinitis, with distinct clinical phenotypes emerging according to allergen type [8]. Indoor perennial allergens, such as house dust mites, cause year-round nasal obstruction, rhinorrhea, and sneezing, often with nocturnal symptoms because of bedroom exposure [8,9]. Pet dander produces perennial symptoms with acute exacerbations upon direct contact [10]. By contrast, grass and weed pollen generate episodic symptoms aligned with pollination periods, and tree pollen frequently provokes early-season disease and can cross-react with food allergens through oral allergy syndrome [11,12]. Geographic and environmental contexts—such as regional vegetation, climate, urbanization, housing, and pet ownership—further modulate exposure intensity and timing, creating location-specific allergic phenotypes; population differences in immune endotypes have also been reported [7,13,14,15].

Although allergic rhinitis studies have reported links between allergen type and symptom patterns, systematic analyses are scarce because most studies examine single allergens or small panels rather than the full spectrum [16]. Accordingly, large-cohort evaluations of CRS symptom profiles by allergen type are lacking. This gap reflects methodological barriers: widespread polysensitization blurs individual allergen effects [17], multiplex IgE testing yields high-dimensional data that challenge conventional analyses [18,19], and demographic or exposure-related confounding can mask true biological associations [20].

To overcome these challenges, we previously applied nonnegative matrix factorization (NMF) to 35-allergen specific-IgE panels, deriving biologically coherent allergen signatures (Mite, Grass/Weed, Pet, and Tree) [21]. These signatures summarized latent exposure-linked patterns and explained a large fraction of sensitization variance in a Korean population, with external relevance to allergic diseases [21]. Building on this framework, we applied a signature-based analysis to a large CRS surgical cohort to examine how allergen sensitization patterns relate to SNOT-22-measured symptom burden. Our objective was to assess whether distinct allergen signatures align with recognizable symptom profiles and to explore key modifiers—such as demographic factors and seasonality—that may shape these relations.

## 2. Materials and Methods

### 2.1. Study Design and Participants

**Figure 1:**
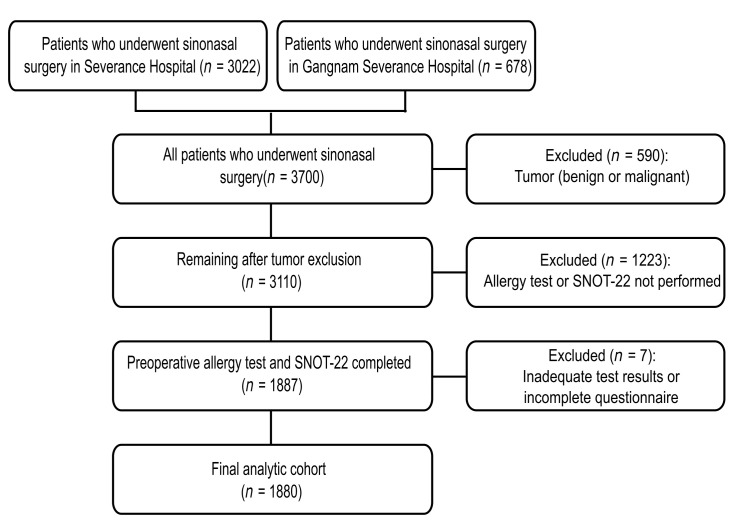
Flowchart of the study cohort. All sinonasal surgeries (*n* = 3700) were screened; tumors were excluded (*n* = 590). Of 3110 eligible patients, 1880 had preoperative Multiple Antigen Simultaneous Test (MAST; 35 allergens) and SNOT-22 within 1 year and comprised the analysis set. SNOT-22, 22-item Sino-Nasal Outcome Test.

We conducted a multicenter retrospective cohort study of patients with CRS, with or without nasal polyposis, who underwent sinonasal surgery at two tertiary hospitals (Severance Hospital and Gangnam Severance Hospital). All patients who underwent sinonasal surgery were screened. The study period was from 1 March 2020 to 27 August 2025. Of the 3700 surgical cases (Severance Hospital, *n* = 3022; Gangnam Severance Hospital, *n* = 678), 590 with benign or malignant sinonasal tumors were excluded, leaving 3110 eligible cases. Among these, 1887 patients who underwent both preoperative allergy testing and completed the SNOT-22 questionnaire were included, and 1223 patients who did not undergo both tests were excluded. An additional seven patients with inadequate test results or incomplete questionnaires were excluded, yielding a final analytical cohort of 1880 patients (Figure 1). All index measures (specific IgE and SNOT-22) were obtained preoperatively within 1 year of surgery; when multiple preoperative assessments were available, the first preoperative evaluation was chosen for subsequent analysis. The study complied with the Declaration of Helsinki and was approved by the Institutional Review Board of Yonsei University College of Medicine (IRB 4-2025-1287). The requirement for informed consent was waived because of the retrospective nature of the study, and all data were anonymized using IRB-approved procedures.

### 2.2. Allergen Testing and Preprocessing

Serum-specific IgE against aeroallergens was quantified using the AdvanSure Allostation Multiple Antigen Simultaneous Test (LG Life Sciences, Seoul, Korea). The clinical panel comprised 35 inhalant allergens (mites, tree/grass/weed pollen, animal dander, fungi, and insects, Table A1), and the results were analyzed using the manufacturer’s class scale (0–6). The mapping of allergens to the 35-feature matrix matched the training matrix used to derive allergen signature loadings H in a previous study [21].

### 2.3. Signature Deconvolution Using the Pretrained H Matrix

Each patient’s 35-dimensional class-scaled Multiple Antigen Simultaneous Test vector (x) was projected onto the pretrained loading matrix H (35 × 4) published by our group (signatures: Mite, Grass/weed, Pet, Tree). Nonnegative signature weights (w; length 4) were estimated using nonnegative least squares, solving minimize ||x − H·w|| subject to w ≥ 0. The dominant signature was defined as the index with the largest weight. Samples with all 35 class-0 entries were labeled nonallergic and assigned w = 0. This deconvolution strategy leverages the portability and biological interpretability of the previously validated H, while ensuring strict comparability to that study’s scale and allergen set.

### 2.4. Outcomes: SNOT-22

Patients completed the SNOT-22. All 22 items (0–5), five prespecified domains (Nasal, Ear/Facial, Sleep, Function, Emotion), and the total score (0–110) were analyzed. Domain scores were computed as item means (0–5) to keep β on the item scale. When multiple visits were present, the index analysis used the first eligible visit, and the sensitivity analyses used mixed-effects models across visits. In the case of pediatric subjects (ages 0–9 years: *n* = 5, 0.27%; ages 10–19 years: *n* = 64, 3.40%), the SNOT-22 instrument was administered by a parent or guardian in conjunction with the child.

### 2.5. Statistical Analysis

SNOT-22 outcomes (items, domain subscores, and total score) were modeled using ordinary least squares regression, with all four allergen signature contributions (Mite, Grass/weed, Pet, and Tree) entered simultaneously as continuous proportions, scaled so that β represents the change per 10-percentage-point increase in a given signature, adjusting for age and sex. Item-level multiplicity was controlled using the Benjamini–Hochberg false discovery rate (*q* < 0.05), whereas domain and total models were summarized with nominal *p*-values and confidence intervals. Descriptive displays included sex-stratified age curves (by decade) and monthly means using the dominant signature.

## 3. Results

### 3.1. Allergen Signatures and Dominant Groups

**Figure 2:**
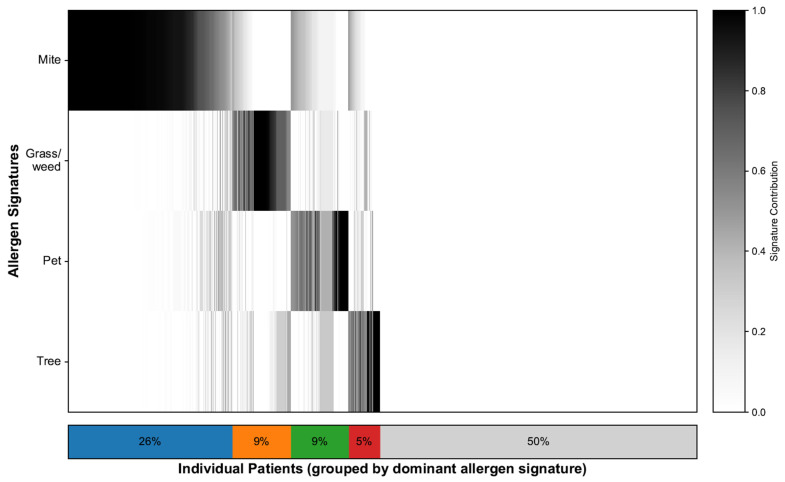
Allergen signature deconvolution and distribution. Heatmap showing 35-allergen sensitization per patient, ordered by dominant signature. The stacked bars summarizes the proportion of patients in each dominant signature group, where blue represents Mite, orange represents Grass/weed, green represents Pet, red represents Tree, and gray represents the Nonallergic group.

To characterize the sensitization landscape of the cohort, we deconvolved each patient’s 35-allergen serum-specific IgE profile, which resulted in continuous contributions to the four allergen signatures and a single dominant assignment for each patient. NMF identified four interpretable signatures: mites, grass/weeds, pets, and trees. Figure 2 illustrates the patient-level contributions, with the stacked bar summarizing the dominant groups: mites (26%), grass/weeds (9%), pets (9%), trees (5%), and nonallergic (50%). The demographic characteristics and SNOT-22 scores of each signature group are shown in Table 1. In line with prior work [21], we additionally flagged a “mixed” profile defined as having ≥2 signatures each ≥25%; this comprised 325 patients (17.3%), whereas 608 patients (32.3%) exhibited a single dominant signature. To minimize misclassification from near-ties and loss of power from dichotomizing polysensitization, all signature–SNOT-22 association models used the continuous signature weights.

### 3.2. Age and Sex Interactions

**Figure 3:**
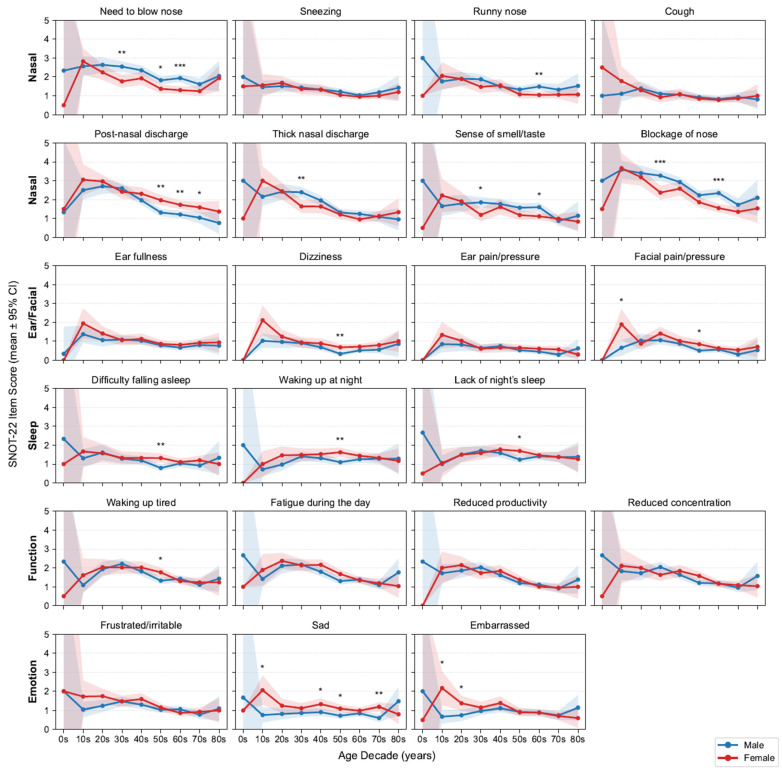
Age- and sex-stratified SNOT-22 item means. Grid of line plots by decade and sex with 95% confidence intervals (CIs); items are grouped by domains. Asterisks above points indicate statistically significant differences between sexes within that age decade (* *p* < 0.05, ** *p* < 0.01, *** *p* < 0.001; false discovery rate [FDR]-corrected). SNOT-22, 22-item Sino-Nasal Outcome Test.

Because SNOT-22 symptom levels vary by age and sex, we first profiled age- and sex-stratified symptom trajectories (Figure 3) and accordingly adjusted for these covariates in all subsequent signature–symptom models. Across the 22 items, mean scores generally declined with age, with the steepest drop from adolescence to early adulthood and then to midlife before plateauing. Regarding nasal symptoms, men generally reported high scores for the need to blow their nose, having a runny nose, thick nasal discharge, loss of smell or taste, and nasal blockage, with significant differences observed in certain age groups. By contrast, women were more likely to report high postnasal discharge scores. No consistent sex differences were noted for sneezing or coughing.

**Figure A1:**
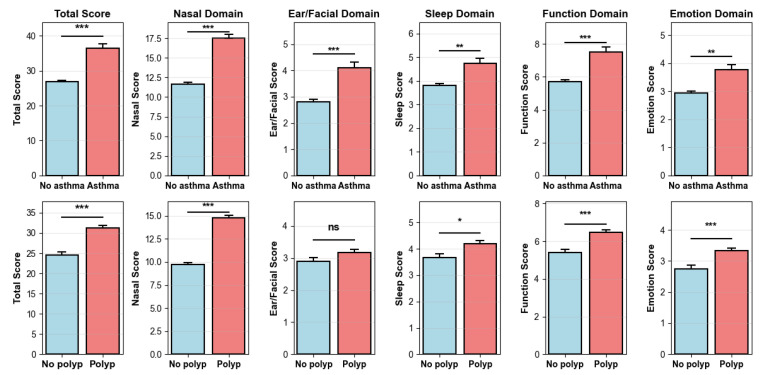
Mean SNOT-22 total and domain scores by asthma (**top**) and nasal polyps (**bottom**). Bars show means with standard error. Significance: *** *p* < 0.001, ** *p* < 0.01, * *p* < 0.05, ns = not significant.

Outside the nasal domain, Ear/Facial scores were low and largely flat across ages, aside from a modest early-adult peak in facial pain/pressure (female predominant). Sleep complaints slightly increased until midlife (30s–50s) and then stabilized or declined; women more frequently reported difficulty falling asleep and waking at night, with significance observed in their 50s. Function (fatigue, productivity, concentration) and Emotion (frustration/irritability, sadness) scores were highest in adolescence and early adulthood and declined thereafter, with an early female predominance that attenuated with age. Building on these demographic patterns, we next examined clinical comorbidities. We report the prevalence of key features—nasal polyps 57.9% and asthma 13.9%. As expected, patients with asthma had higher SNOT-22 total and all domain scores than those without asthma; patients with nasal polyps had higher total and domain scores except for the Ear/Facial domain, which did not differ significantly (Figure A1). These domain-specific trajectories aligned with clinical expectations and motivated adjustment for age, sex, asthma and nasal polyps in the following association analyses.

### 3.3. Signature–Symptom Associations

**Figure 4:**
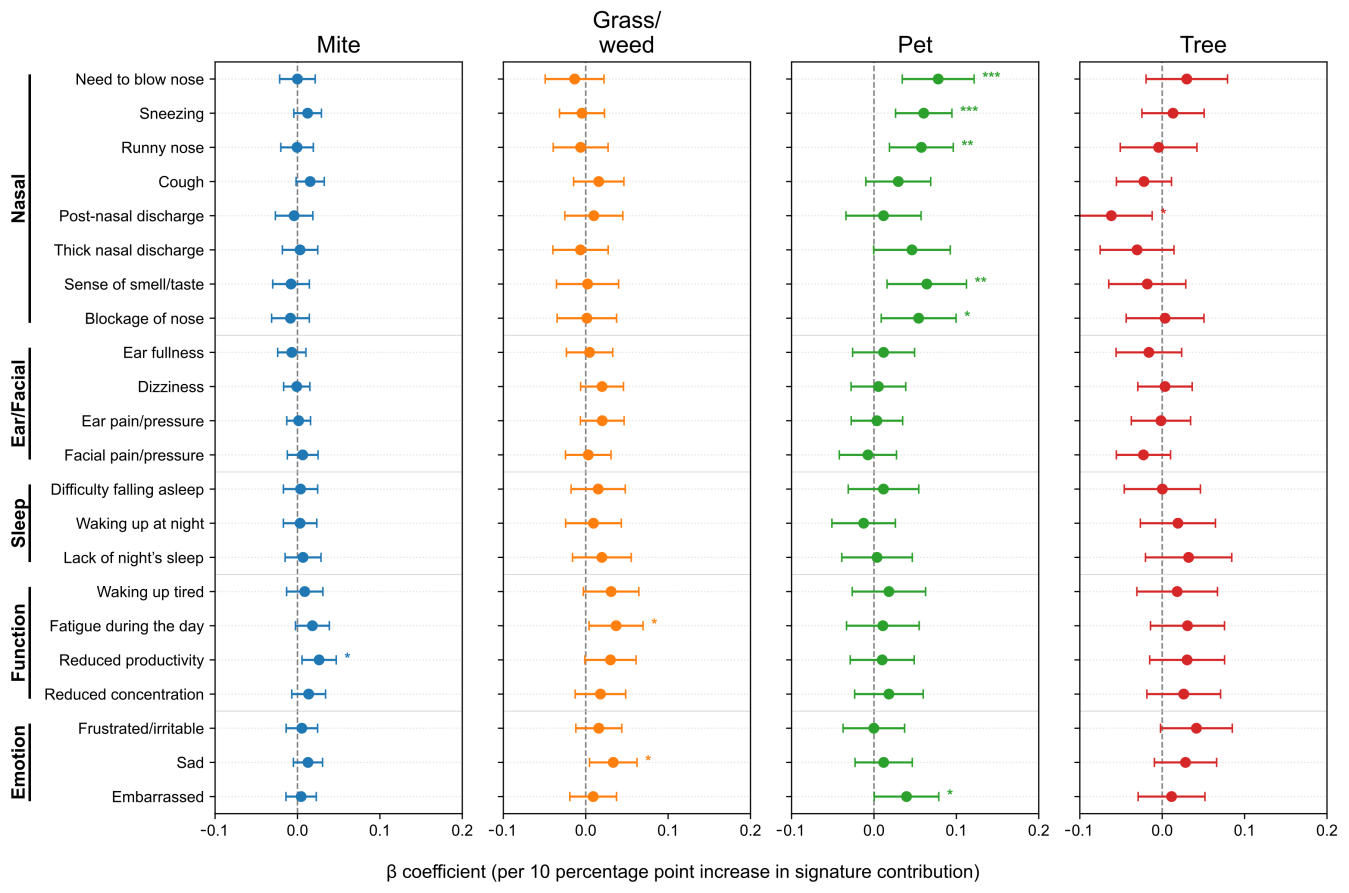
Associations between allergen signatures and individual SNOT-22 items. Forest plots show adjusted β per 10-percentage-point increase in signature contribution with 95% confidence intervals (CIs). Items are grouped by domain; intervals crossing zero indicate non-significance. SNOT-22, 22-item Sino-Nasal Outcome Test. Statistical significance is indicated by asterisks: *p* < 0.05 (*), *p* < 0.01 (**), and *p* < 0.001 (***).

**Figure A2:**
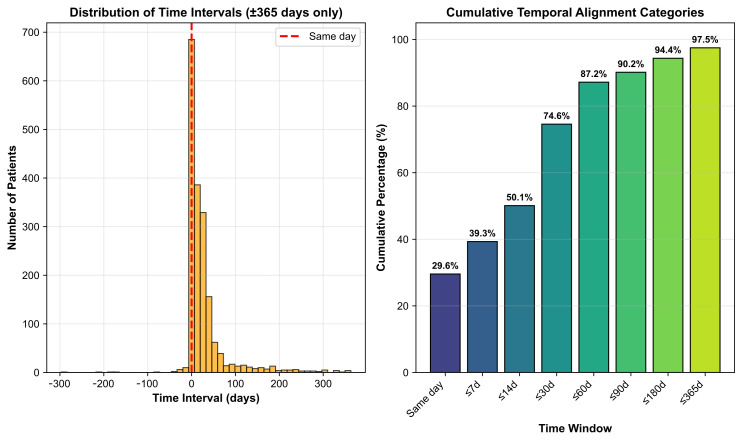
Temporal alignment between SNOT-22 and specific IgE testing. (**Left**): Histogram of the time interval (days) between SNOT-22 and IgE testing for pairs within ±365 days; positive values indicate SNOT-22 obtained after IgE. The red dashed line marks same-day measurement. (**Right**): Cumulative percentage of patient-pairs falling within prespecified windows with bar labels showing the cumulative proportion.

**Figure A3:**
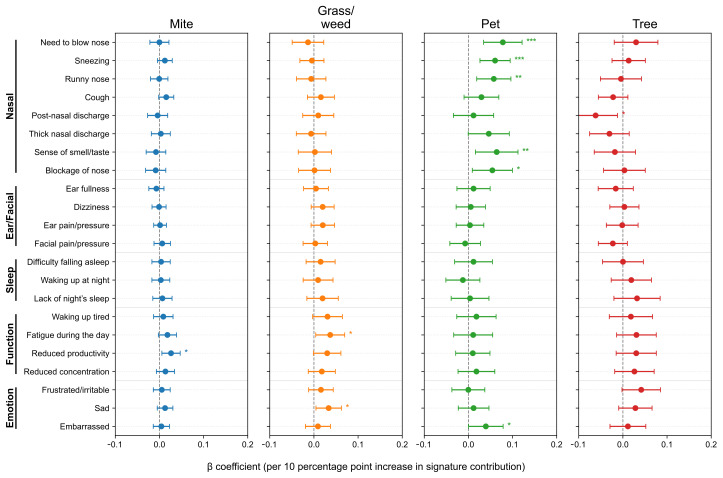
Associations between allergen signatures and individual SNOT-22 items in the subset of patients whose SNOT-22 and IgE measurements were obtained within 30 days. Forest plots show age- and sex-adjusted β per 10-percentage-point increase in signature contribution with 95% confidence intervals (CIs). Items are grouped by domain; intervals crossing zero indicate non-significance. SNOT-22, 22-item Sino-Nasal Outcome Test. Statistical significance is indicated by asterisks: *p* < 0.05 (*), *p* < 0.01 (**), and *p* < 0.001 (***).

After adjusting for age, sex, asthma and nasal polyps, we further analyzed SNOT-22 scores according to allergen signature. At the item level (Figure 4), the Pet signature showed the strongest and most consistent associations with the Nasal domain items—need to blow nose (β = 0.0648, *p* = 7.0 × 10^−4^), sneezing (0.0607, *p* = 8.9 × 10^−5^), runny nose (0.0599, *p* = 4.4 × 10^−4^), and nasal blockage (0.0456, *p* = 0.025), with a positive association for embarrassment (0.0361, *p* = 0.039). The Mite signature was selectively associated with Function/Emotion items–daytime fatigue (0.0202, *p* = 0.025), reduced productivity (0.0246, *p* = 0.0075), and sadness (0.0178, *p* = 0.0289). The Grass/weed signature similarly related to waking up tired (0.0377, *p* = 0.0169), daytime fatigue (0.0399, *p* = 0.0082), reduced productivity (0.0287, *p* = 0.0470), and sadness (0.0393, *p* = 0.0040). The Tree signature showed no significant item-level associations (*p* < 0.05). To minimize seasonal misalignment, we re-estimated models restricting to pairs with SNOT-22 and IgE obtained within 30 days (*n* = 1402, 74.6%, Figure A2). The direction and significance patterns were essentially unchanged: the Pet signature remained significantly associated with Nasal items, Mite with reduced productivity, and Grass/weed with sadness (Figure A3). These results indicate seasonality-related timing differences are unlikely to drive our findings.

**Figure 5:**
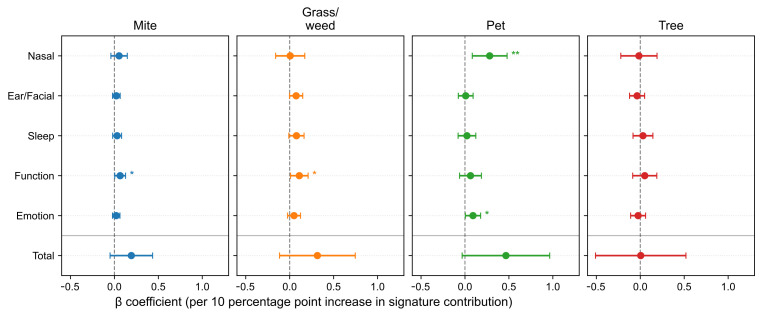
Associations between allergen signatures and SNOT-22 domain subscores and total scores. Forest plots report adjusted β per 10-percentage-point increase with 95% confidence intervals (CIs). A horizontal divider separates domain subscores from the total score. SNOT-22, 22-item Sino-Nasal Outcome Test. Statistical significance is indicated by asterisks: *p* < 0.05 (*), *p* < 0.01 (**).

Models adjusted for age, sex, and comorbidities yielded selective associations at the domain level (Figure 5). The Pet signature was associated with the Nasal domain subscore (β = 0.2811, *p* = 0.00547) and Emotion domain subscore (β = 0.0912, *p* = 0.0400). Both the Mite and Grass/weed signatures were associated with the Function domain (β = 0.0654, *p* = 0.0374; β = 0.1097, *p* = 0.0318, respectively). The Tree signature showed no significant domain-level associations (*p* < 0.05).

### 3.4. Seasonal Patterns

**Figure 6:**
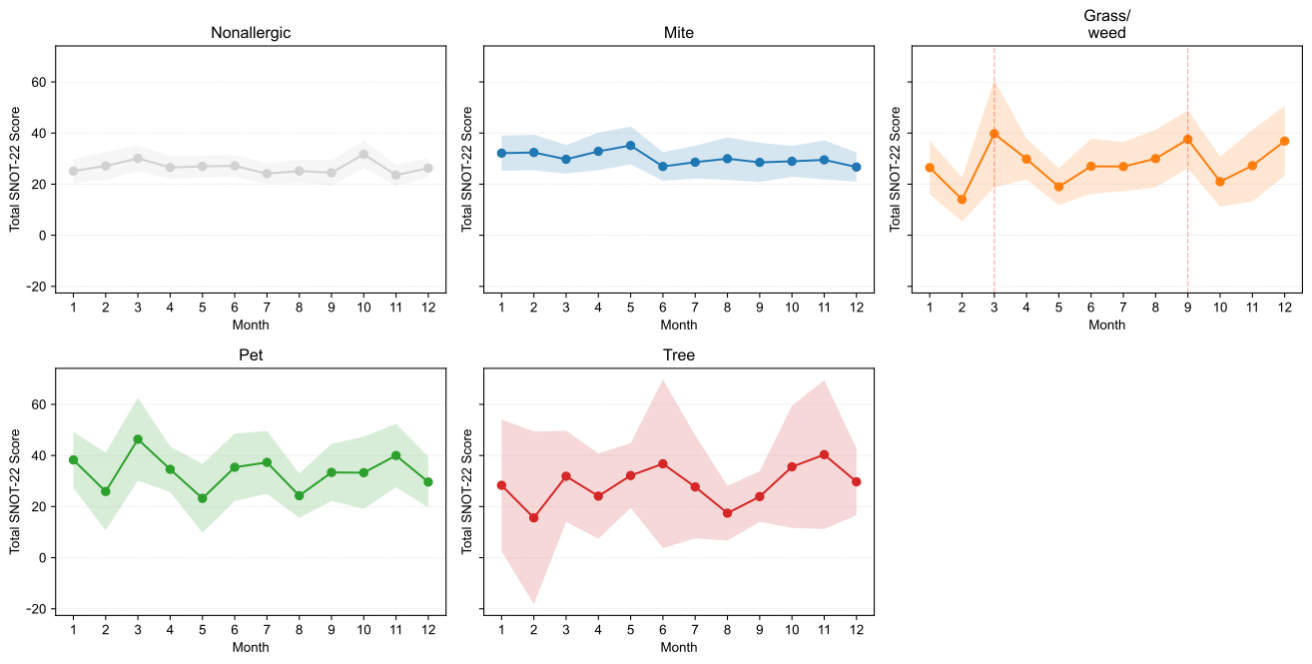
Monthly total SNOT-22 scores by dominant signature. Small multiples display monthly means with 95% confidence intervals (CIs) for Mite, Grass/weed, Pet, Tree, and Nonallergic groups, enabling visual comparison of seasonal symptom patterns across sensitization profiles. SNOT-22, 22-item Sino-Nasal Outcome Test.

The average monthly SNOT-22 scores were compiled based on the dominant allergen signature (Figure 6). Hereafter, “seasonal variation” is defined as the coefficient of variation (CV) of monthly mean SNOT-22 scores—(standard deviation of monthly averages ÷ annual average) × 100%—with larger CV indicating greater seasonality. Patients without allergic sensitization exhibited minimal seasonal changes (annual average 26.52; CV 9.06%). Patients with mite sensitization showed winter–spring predominance with low variability (average 30.13; CV 8.39%). Patients with grass/weed sensitization had the greatest seasonal variation (average 27.97; CV 27.41%), with a bimodal pattern peaking in March (39.73, +42% compared with the average) and September (37.56, +34%). Patients with pet-dominant sensitization had the highest absolute symptom burden throughout the year (average 33.46; CV 20.41%), with peaks in January (38.28), March (46.35), July (37.31), and November (40.00) and the lowest in May (23.22). Patients with tree-dominant sensitization showed notable variability (average 28.62; CV 26.10%), with peaks in June (36.75) and October–November (35.57–40.33) and a low in February (15.60). Overall, relative seasonal variation was most pronounced for grass/weed and tree sensitizations, moderate for pet sensitization, and minimal for mite and nonallergic groups, whereas absolute symptom burden ranked as pet > mite > tree ≈ grass/weed > nonallergic, aligning with exposure ecology.

## 4. Discussion

In this extensive, real-world surgical cohort, IgE-derived allergen signatures exhibited differential associations with patient-reported symptom burden measured by the SNOT-22. After adjusting for age and sex, whose significant baseline effects were initially delineated, signature–symptom associations followed coherent, exposure-linked patterns: the Pet signature was most strongly associated with nasal items and the Nasal domain and was the only signature linked to a high total SNOT-22 score; Mite signature was associated with the Function domain, alongside item-level increases in daytime fatigue and productivity loss; Grass/weed signature was associated with the Function and Emotion domains, with item-level increases in fatigue, productivity loss, and sadness; and Tree signature showed no significant associations. Seasonal analyses further supported ecological plausibility: grass/weed and tree sensitizations exhibited the largest relative variation across months, mite sensitization showed minimal variation with winter–spring predominance, and pet sensitization carried the highest year-round absolute burden.

Multiple factors influence CRS immunopathogenesis and phenotype. The link between allergies and CRS has received considerable attention but remains controversial. Patients with atopy are more likely to develop CRS associated with nasal polyps [22]. By contrast, overall disease severity is not consistently associated with atopic status in patients with CRS [23,24]. Notably, little is known about whether PROMs differ according to allergic sensitization patterns in patients with CRS. Therefore, we stratified patients with CRS based on allergen sensitization patterns using a previously validated NMF model and revealed the allergen signature-associated symptom profiles. To the best of our knowledge, this is the first study to delineate PROMs in CRS based on NMF-derived allergen signatures. Our findings indicate potential differential effects of sensitization to specific allergens on CRS phenotypes.

This study extends the literature on allergic rhinitis to the CRS setting and aids in parsing symptom heterogeneity not captured by anatomy alone. A strong pet-associated nasal signal, coupled with greater smell or taste impairment at the item level, aligns with continuous indoor exposure and proximity-driven mucosal inflammation. By contrast, Mite and Grass/weed associations with Function (and for Grass/weed, Emotion) suggest that perennial bedroom exposure and seasonal pollination-period exposure may extend beyond rhinologic complaints to include sleep fragmentation, daytime fatigue, and affective symptoms—domains that patients often prioritize in clinical encounters. Item-level inference was controlled for multiplicity, underscoring that these are not chance findings dispersed across the 22 questions but rather clustered, domain-consistent effects.

Employing the previously substantiated NMF basis with the Dog–Pet loading defined as H_Dog,Pet_ ≈ 7.96 [21], a transition of Dog-specific IgE from class 0 to class 6 suggests a corresponding alteration in the Pet-signature quantified as Δw_Pet_ ≈ 6/7.96 = 0.754, assuming local linearity and holding other signatures constant. Utilizing our regression scaling per 10-percentage-point increment, the forecasted alterations in SNOT-22 are as follows: Nasal domain + 2.12 points and Emotion + 0.69 points. These values fall short of the documented minimally clinically important differences (MCIDs)—4 points for the Nasal domain and 1 point for Emotion—and therefore do not, in isolation, signify a clinically significant alteration at the individual patient level [25]. It is crucial to note that MCIDs were devised to capture post-treatment variations (medical or surgical) rather than cross-sectional disparities associated with sensitization. These domain-specific effects remain relevant for guidance and preventive measures (e.g., reducing indoor pet exposure, proactive planning) and may accumulate alongside seasonal, comorbid, and therapeutic elements to provide substantial benefits.

In clinical practice, a signature-informed approach can enhance counseling and establish realistic expectations. Patients with pet-dominant allergies may benefit from targeted indoor exposure mitigation strategies, such as bedroom exclusion, HEPA filtration, and strict hygiene, with the understanding that nasal symptoms significantly contribute to the overall burden. Conversely, patients with mite-dominant allergies may require bedroom humidity control and mattress or pillow encasing, with particular attention to functional recovery, including fatigue and productivity. Patients with grass/weed-dominant allergies may benefit from anticipatory management during spring and fall peaks, including preseason pharmacotherapy and scheduling activities during low-pollen periods, with a focus on mood and function. Beyond counseling, allergen signatures could enhance the selection of candidates for allergen immunotherapy or environmental interventions and provide domain-specific endpoints for monitoring perioperative and longitudinal care.

The strengths of this study include a large multisite cohort; data-driven deconvolution of polysensitization using a pretrained, biologically interpretable NMF basis; item-level modeling with age and sex adjustment and false discovery rate control; and seasonality analysis aligned with exposure ecology. However, certain limitations warrant consideration: the retrospective design and surgical inclusion criteria may limit generalizability to non-surgical CRS. We pre-specified a surgical cohort to ensure clear index encounters and uniform availability of IgE testing and SNOT-22 in a retrospective record-based design, making it unfeasible to assemble a comparable non-surgical CRS cohort from routine records due to heterogeneous referral patterns, inconsistent SNOT-22 acquisition, and incomplete allergy testing. Consequently, enrichment for greater baseline symptom burden in surgical candidates could bias the direction or magnitude of signature–PROM associations, potentially inflating associations for perennial/type-2–linked signatures or, conversely, attenuating contrasts via ceiling effects. We therefore interpret our findings as most applicable to surgical CRS and explicitly call for validation in prospectively enrolled non-surgical cohorts. Second, we were unable to discern whether the associations between signatures and symptoms predominantly indicate comorbid allergic rhinitis or a CRS-specific influence of sensitization. A longitudinal study that includes a comparator group consisting solely of individuals with allergic rhinitis is necessary. Third, some potential confounders were not reliably ascertainable in the medical records—specifically nasal corticosteroid/systemic steroid use and prior endoscopic sinus surgery—owing to multi-institutional care and variable documentation; as a result, we could not adjust for them across the cohort, and residual confounding may persist despite adjustment for age, sex, and comorbidities. Fourth, the seasonal analysis used dominant groups rather than continuous signature contributions. Fifth, small monthly sample sizes in some signatures, particularly Tree, increased uncertainty. Lastly, the current cohort are solely representative of Korean populations, thus potentially restricting their applicability to other ancestral groups and environmental exposures. Future research should prospectively test whether signatures can predict responses to environmental control or allergen immunotherapy, integrate objective inflammatory and olfactory measures, and validate portability across regions and assay platforms.

In conclusion, allergen signatures derived from clinical IgE panels elucidate the symptom heterogeneity captured by the SNOT-22 in CRS, and incorporating these signatures into routine assessments may support personalized, domain-focused management.

## Figures and Tables

**Table 1:** Demographic and SNOT-22 scores of each allergen signature group.

	Nonallergic	Mite	Grass/Weed	Pet	Tree	*p*-Value
N	947	491	175	173	94	
Age (years), mean ± SD	55.3 ± 16.3	49.7 ± 17.0	58.5 ± 13.1	46.6 ± 19.0	51.9 ± 15.6	<0.001
Female, *n* (%)	521 (55.0%)	159 (32.4%)	63 (36.0%)	75 (43.4%)	36 (38.3%)	<0.001
SNOT-22 scores, mean ± SD						
Nasal	11.5 ± 8.8	13.4 ± 9.3	11.3 ± 8.6	15.8 ± 9.9	12.6 ± 9.3	<0.001
Ear/Facial	2.9 ± 3.6	3.1 ± 3.8	3.0 ± 3.9	3.4 ± 3.8	2.6 ± 3.4	0.348
Sleep	3.8 ± 4.1	3.9 ± 4.1	4.2 ± 4.2	4.3 ± 4.3	4.0 ± 4.2	0.658
Function	5.4 ± 5.2	6.4 ± 5.6	6.3 ± 5.2	7.2 ± 5.9	6.2 ± 5.6	<0.001
Emotion	2.8 ± 3.2	3.2 ± 3.4	3.3 ± 3.3	3.6 ± 3.5	3.0 ± 3.2	0.020
Total score	26.5 ± 20.1	30.1 ± 21.2	28.1 ± 21.1	34.3 ± 22.2	28.4 ± 20.9	<0.001

SNOT-22, 22-item Sino-Nasal Outcome Test; SD, standard deviation.

## Data Availability

Due to patient privacy considerations and institutional restrictions, the datasets generated and/or analyzed during this study are not publicly available but may be obtained from the corresponding author upon reasonable request and with appropriate approvals.

## Abbreviations

The following abbreviations are used in this manuscript:

CRS	Chronic rhinosinusitis
PROMs	Patient-reported outcome measures
SNOT-22	22-item Sino-Nasal Outcome Test
NMF	Nonnegative matrix factorization
CV	Coefficient of variation

## Appendix A

**Table A1:** H Table for the Four NMF-Derived Allergen Signatures.

	Comp1	Comp2	Comp3	Comp4
Acacia	0	2.656	0	0.404
Alternaria	0.505	0.134	1.674	1.203
Ash Tree	0	3.067	0	0.585
Aspergillus	0.315	0.27	0.476	0.293
Bermuda Grass	0.051	6.37	0	0
Birch	0	0	0.09	11.524
Cat	0	0	16.083	0
Cedar	0	2.237	0	0.792
Cladosporium	0.256	0.271	0.341	0.242
Cockroach	0.991	2.181	0	0
Crab	1.182	0.395	0	0
Dandelion	0.08	3.59	0.187	0.712
*D. farinae*	18.583	0.48	0	0
*D. pteronyssinus*	16.811	0	0.442	0
Dog	0.218	0.246	7.956	0
Egg White	0.345	0.179	0.489	0.718
Goldenrod	0.08	3.789	0.242	0.764
Hazelnut	0	1.698	0	2.56
Hop	0.178	2.116	0.22	1.694
House Dust	10.942	0.435	2.043	0.015
Mackerel	0.05	0.039	0.06	0.06
Milk	0.684	0.183	0.338	0.02
Mugwort	0	4.298	0.112	0.759
Oak	0	4.191	0.136	2.265
Orchard Grass	0	6.557	0	0
Peach	0	0.432	0	9.689
Penicillium	0.291	0.223	0.28	0.08
Pigweed	0	4.754	0	0.318
Ragweed	0	5.372	0	0.338
Russian Thistle	0	6.46	0	0
Rye	0.027	7.992	0.06	0
Shrimp	1.128	0.269	0.05	0.11
Soybean	0	1.543	0	0.416
Sycamore	0	3.399	0	1.073
Timothy Grass	0	7.057	0.062	0
